# MRI reveals hemodynamic changes with acute maternal hyperoxygenation in human fetuses with and without congenital heart disease

**DOI:** 10.1186/1532-429X-17-S1-O55

**Published:** 2015-02-03

**Authors:** Prashob Porayette, Liqun Sun, Edgar Jaeggi, Lars Grosse-Wortmann, Shi-Joon Yoo, Edward J Hickey, Steven Miller, Christopher Macgowan, Mike Seed

**Affiliations:** 1 Paediatric Cardiology, The Hospital for Sick Children, Toronto, ON, Canada; 2 Diagnostic Imaging, The Hospital for Sick Children, Toronto, ON, Canada; 3 Physiology & Experimental Medicine, The Hospital for Sick Children, Toronto, ON, Canada; 4 Cardiovascular Surgery, The Hospital for Sick Children, Toronto, ON, Canada; 5 Neurology, The Hospital for Sick Children, Toronto, ON, Canada

## Background

Maternal hyperoxygenation (MH) has been used for intrauterine growth restriction and proposed as a way to improve ventricular growth in the setting of congenital heart disease (CHD)[1,2]. Fetal lamb experiments reveal increases in the SaO_2_ of umbilical venous (UV) blood and a reduction in pulmonary vascular resistance during MH [3]. Doppler suggests that MH increases the human fetal pulmonary blood flow (PBF) [4]. The combination of fetal phase contrast (PC) MRI and MR oximetry using T2 mapping offers the potential for a comprehensive hemodynamic assessment of late gestation fetal circulation [5]. We investigated the physiologic impact of MH in human fetuses with and without CHD using MRI to explore the potential therapeutic benefits of chronic MH.

## Methods

We examined 17 normal human fetuses [mean gestational age (GA) of 37.3 wks; SD ± 1 wk] and 20 fetuses with CHD (mean GA of 36.2 wks; SD ± 1 wk) on a 1.5T system (Siemens Avanto, Erlangen, Germany) after hospital IRB approval. Flows were measured in major fetal vessels using PC MRI and indexed to fetal weight along with T2 of UV blood according to our previously published technique [5,6]. According to the Luz-Meiboom equation [^7^] the T2 relaxation of blood is proportional to its O2 saturation. The measurements were repeated during MH (12 L/min of O2 via a non-rebreather mask, FiO_2_ ∼70%). Results were compared using Student's *t*-test, with results with p-value ≤ 0.05 considered statistically significant.

## Results

**Figure 1:**
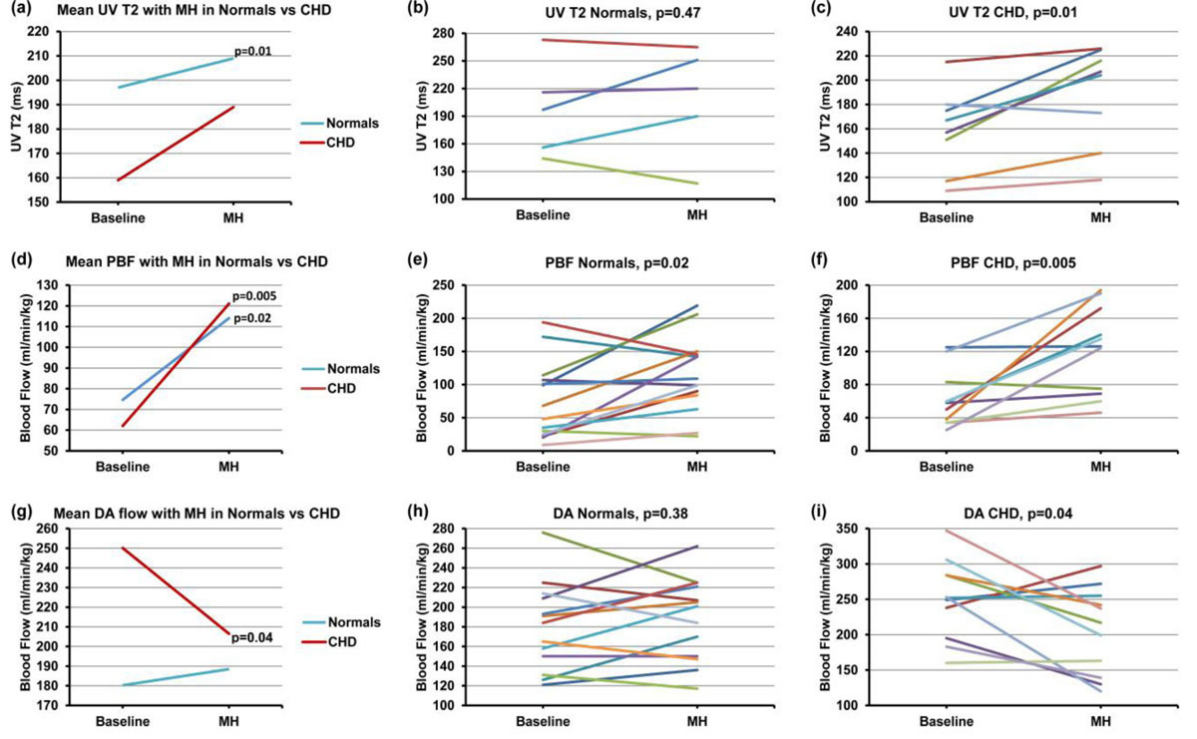
**Physiologic impact of maternal hyperoxygenation (MH) in late gestation human fetuses with and without congenital heart disease (CHD).** At baseline, the umbilical venous (UV) T2 (a) was lower in CHD fetuses than in normals. Although the UV T2 did not change significantly with MH in normals (b), we observed a significant increase in UV T2 in CHD fetuses with MH (p=0.01) (c). Both groups showed a statistically significant increase in pulmonary blood flow (PBF) during MH (d), which was more dramatic in fetuses with CHD (p=0.005) (e, f). While there was a significant reduction in ductus arteriosus (DA) flow in CHD fetuses during MH (p=0.04) (g,i), this was not present in normal fetuses (h). p ≤ 0.05 statistically significant.

At baseline, the UV T2 was lower in CHD fetuses than in normals. Although UV T2 did not change significantly with MH in normals, we observed a significant increase in UV T2 in CHD fetuses with MH (p=0.01, Fig. 1, Table 1). Both groups showed a statistically significant increase in PBF during MH but was more dramatic in CHD fetuses (p=0.005). While there was a significant reduction in ductus arteriosus (DA) flow in CHD fetuses during MH (p=0.04), this was not present in normals. There was no significant difference in blood flow in any of the other major vessels.

**Table 1:** Average blood flow and T2 with maternal hyperoxygenation in human fetuses with and without congenital heart disease.

Normals				CHD			
	**Baseline**	**MH**	***p***		**Baseline**	**MH**	***p***

**UV T2 (ms, n=5)**	197.2	208.6	0.47	**UV T2 (ms, n=8)**	158.88	188.63	**0.01**

**PBF (ml/kg/min, n=14)**	74.57	114.1	**0.02**	**PBF (ml/kg/min, n=11)**	62.36	121	**0.005**

**DA (ml/kg/min, n=13)**	180.23	188.46	0.38	**DA (ml/kg/min, n=11)**	250.09	206.45	**0.04**

CHD: congenital heart disease, MH: maternal hyperoxygenation, UV: umbilical venous, PBF: pulmonary blood flow, DA: ductus arteriosus, p ≤ 0.05 statistically significant

## Conclusions

The reason for lower O2 saturations in the UV of fetuses with CHD is uncertain, but may reflect abnormal placental and/or fetal cardiovascular function. The lower position of UV blood saturation on the O2 dissociation curve of hemoglobin may explain the higher uptake of O2 from maternal plasma in CHD fetuses. The expected increase in PBF with MH was observed in both groups. This increase in PBF also explains the lower DA flow in CHD fetuses with MH who had significantly higher DA flow compared to normal at baseline (p=0.002). This study suggests that fetal MR can be used to assess hemodynamic changes resulting from MH and could provide useful additional fetal monitoring when MH is being used for therapy.

## Funding

This work was performed with support from the Labatt Family Heart Centre Innovations Fund.
